# Ocular Manifestations Leading to the Diagnosis of Ichthyosis: A Case Report

**DOI:** 10.7759/cureus.80023

**Published:** 2025-03-04

**Authors:** Joobin Khadamy

**Affiliations:** 1 Ophthalmology, Skellefteå Eye Clinic, Skellefteå, SWE; 2 Ophthalmology, University Hospital of Umeå, Umeå, SWE

**Keywords:** corneal erosions, corneal nerves, genodermatosis, ichthyosis, ocular manifestations, ocular surface disease, recurrent corneal erosions, steroid sulfatase deficiency, stromal opacities, x-linked ichthyosis

## Abstract

Ichthyosis, a group of genetic disorders of keratinization, often extends beyond the skin to affect mucosal surfaces, including the ocular surface. This case report of a 15-year-old male illustrates the critical interplay between dermatological conditions and ocular health. Initially treated for chronic eczema, the patient presented with recurrent ocular pain, photophobia, and corneal abnormalities including punctate epithelial erosions, punctate deep stromal opacities, and prominent corneal nerves. A skin biopsy revealed orthokeratosis and a normal granular layer, a key finding supporting a diagnosis of an ichthyosis variant, likely X-linked ichthyosis (XLI), over ichthyosis vulgaris. The ocular manifestations stemming from structural corneal basement membrane abnormalities were pivotal in uncovering the underlying systemic disorder. This case underscores how dermatological diseases can impact ocular surface integrity, emphasizing the need for multidisciplinary care and early recognition of ocular signs to prevent long-term complications like corneal scarring and vision impairment.

## Introduction

Ichthyosis encompasses a group of genetic disorders characterized by defective epidermal differentiation and keratinization, resulting in diffuse skin scaling reminiscent of fish scales - aptly reflected in its Greek root "ichthys," meaning "fish." With a prevalence ranging from one in 250 for common variants like ichthyosis vulgaris, and approximately one in 2,000 to one in 6,000 males affected by the rarer X-linked ichthyosis (XLI), these conditions display marked demographic patterns, notably a male predominance in XLI due to its X-linked recessive inheritance [1,2]. Beyond their cutaneous hallmark, severe forms of ichthyosis can impair mucosal surfaces, including the ocular surface, leading to complications such as keratopathy, dry eye syndrome, and recurrent corneal erosions [2,3]. These ocular manifestations often compromise quality of life and pose significant diagnostic and therapeutic challenges. Although deep stromal opacities are a hallmark of XLI, they remain infrequently captured in visual documentation, underscoring the value of detailed case reports in enriching the clinical understanding of this condition [4]. Herein, we present the case of an adolescent male whose recurrent punctate epithelial keratopathy unmasked underlying ichthyosis, illuminating the critical interplay between systemic dermatological disorders and ocular health [1-3].

## Case presentation

A 15-year-old male was referred to the ophthalmology clinic by his primary care physician to evaluate recurrent episodes of bilateral ocular discomfort and pain, particularly at night. These episodes were associated with photophobia and difficulty opening his eyes upon waking. The symptoms typically resolved spontaneously within hours. At follow-up in the primary ophthalmology clinic, the recurrence of symptoms and punctate elevated corneal erosions led to a referral to a tertiary university eye hospital for further evaluation. Consent from the patient’s guardians was obtained for the publication of this report.

**Figure 1:**
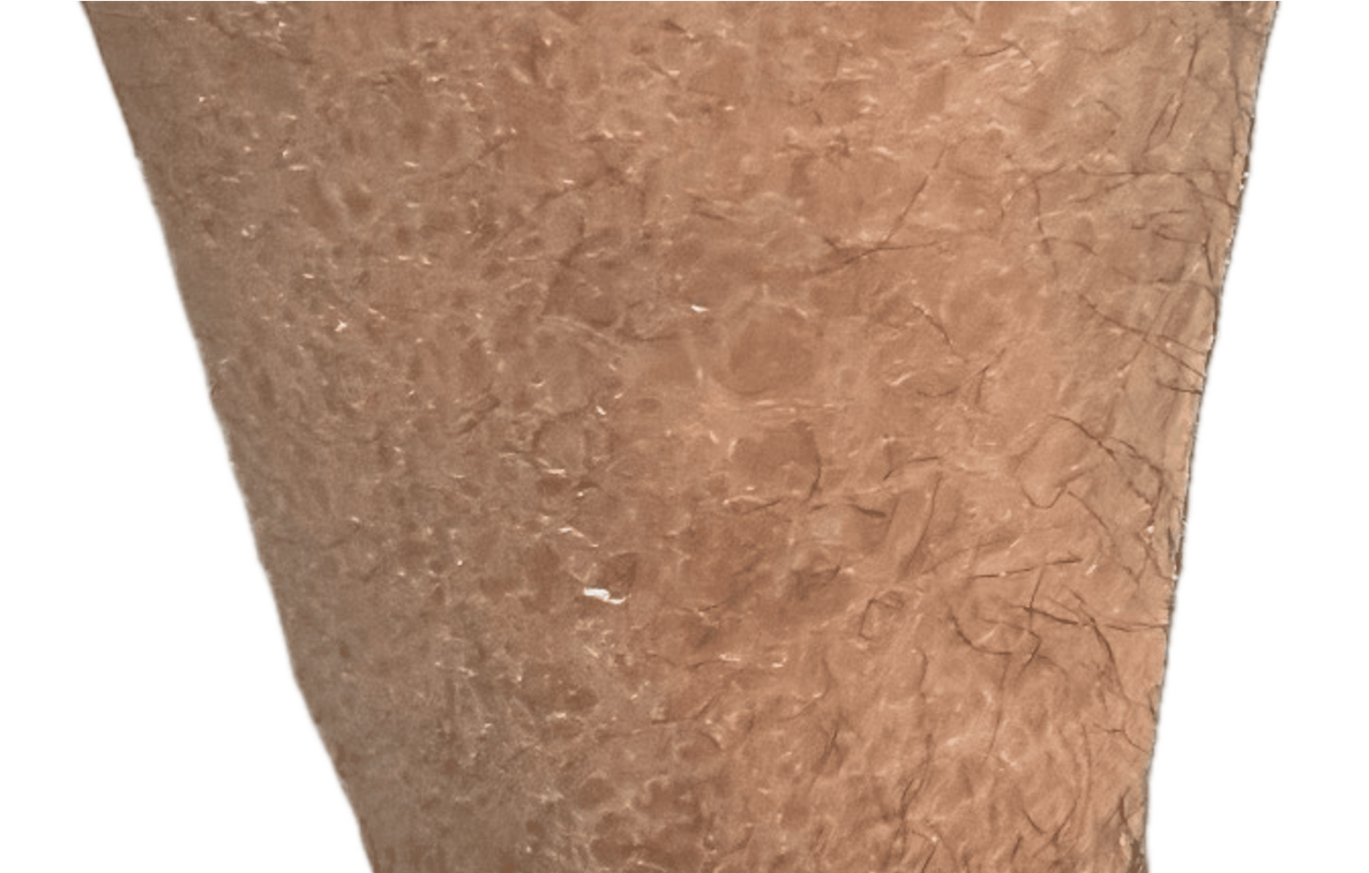
Polygonal, dark, scaly patches with thickened plaques on the lower limb The scales are characteristic of ichthyosis, with persistent skin involvement despite treatment. These findings were previously misdiagnosed as chronic eczema.

Upon deeper exploration at the university eye hospital, the patient’s medical history revealed a long-standing diagnosis of chronic eczema since early childhood. There was no reported family history of similar skin or eye conditions. Despite multiple treatments, his dry, scaly skin persisted, primarily affecting the lower limbs and trunk, with flexural areas showing milder involvement (Figure 1).

**Figure 2:**
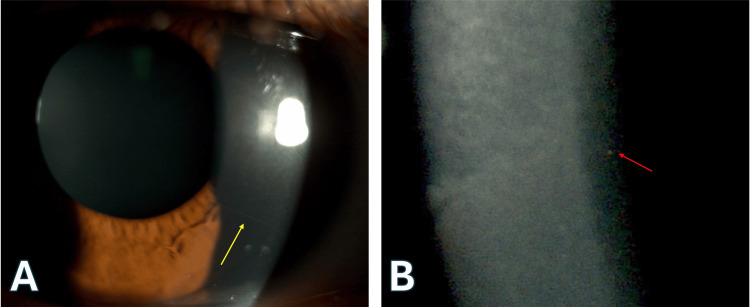
Prominent corneal nerve and punctate deep stromal opacities A: Prominent corneal nerve (yellow arrow). B: Punctate deep stromal opacities (red arrow).

Ophthalmologic examination revealed normal visual acuity and intraocular pressure bilaterally. However, corneal examination showed prominent corneal nerves (Figure 2A) and deep stromal punctate changes (Figure 2B) without active epithelial erosions, suggesting a quiescent phase of the disease. Additionally, pigmented endothelial deposits were noted in both eyes. Eyelid examination revealed a normal position with no ectropion or lagophthalmos, although the skin around the eyes was dry. Tear film assessment demonstrated reduced tear break-up time, indicating ocular surface dryness.

**Figure 3:**
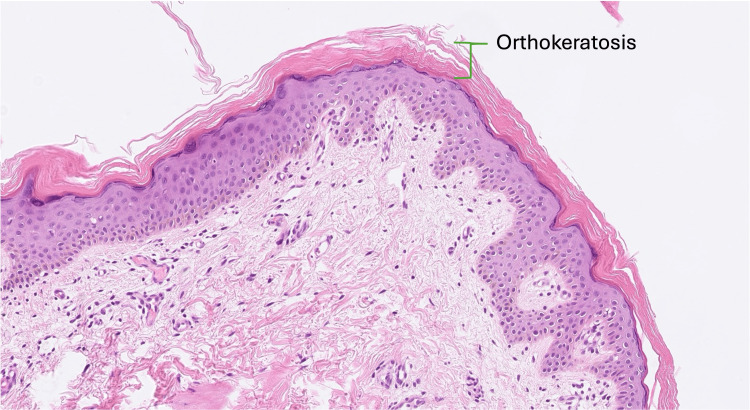
Histopathology of skin biopsy in ichthyosis Histopathology of skin biopsy stained with hematoxylin and eosin (H&E) revealed orthokeratosis (thickened stratum corneum without retained nuclei) and a normal granular layer consistent with X-linked ichthyosis (XLI). There was no evidence of spongiosis or significant inflammatory infiltrate, excluding concurrent inflammatory dermatosis.

Given the ophthalmic findings and the patient’s skin presentation, which resembled ichthyosis, he was referred to a dermatologist for further evaluation. A skin biopsy taken from the lower limb confirmed hyperkeratosis (orthokeratosis) without parakeratosis (Figure 3). The granular cell layer was normal, and the absence of spongiosis or inflammation supported a diagnosis of XLI rather than ichthyosis vulgaris or eczema. 

Deep stromal corneal opacities, a characteristic finding in XLI, and the skin biopsy results supported the diagnosis.

To manage ocular surface dryness, the patient was treated with preservative-free artificial tears during the day and lubricating ointment at night. Although genetic testing was recommended to confirm XLI, the patient was unfortunately lost to follow-up before further investigation could be completed. A referral was made to a dermatologist for systemic management of ichthyosis, with advice for regular emollient application to prevent exacerbation of skin tightness and promote eyelid mobility.

## Discussion

This case illustrates the significant impact of systemic dermatological conditions like XLI on ocular surface health. Individuals with ichthyosis frequently experience dryness and lagophthalmos, making them more susceptible to chronic epithelial stress, recurring erosions, and secondary infections. XLI, an X-linked recessive disorder caused by a deficiency in steroid sulfatase (STS), manifests with dry, scaly skin and ocular findings including corneal erosions, deep stromal opacities, and prominent corneal nerves. Although prominent corneal nerves can occur in atopic conditions linked to ichthyosis vulgaris, the deep stromal opacities observed here are characteristic of XLI, occurring in 10-50% of affected individuals and often presenting in adolescence or early adulthood [2-6].

In this patient, the recurrent episodes of acute ocular pain were likely associated with corneal changes involving the subepithelial and anterior stromal layers, which resulted in an irregular overlying corneal epithelium. Histopathologic studies suggest that these opacities stem from cholesterol sulfate accumulation due to STS deficiency, thickening the corneal epithelial basement membrane with irregular extensions into Bowman’s layer [7,8]. This accumulation disrupts lipid composition in the cornea, contributing to stromal opacities and predisposing the surface to erosions, as seen in this 15-year-old patient. These changes caused the patient’s episodes of acute ocular discomfort and photophobia [7-10].

The initial misdiagnosis of eczema underscores the importance of distinguishing between chronic inflammatory skin disorders and genetic keratinization disorders like ichthyosis. XLI’s cutaneous presentation - polygonal, dark scales on extensor surfaces sparing palms and soles - differs from the fine, whitish scales of ichthyosis vulgaris, yet overlaps can complicate diagnosis [2,3]. The differential diagnosis of ichthyosis and its ocular manifestations is essential for accurate management. Table 1 outlines key differences between ichthyosis subtypes and their respective ocular findings [2,3]. Although ichthyosis vulgaris was initially considered, the ocular findings and the histopathological results strongly supported a diagnosis of XLI. Histopathological evidence of a normal granular layer is a hallmark of XLI that distinguishes it from ichthyosis vulgaris, which typically lacks this layer. Unfortunately, the patient was lost to follow-up before genetic testing could be performed, which would have provided a definitive diagnosis.

**Table 1:** Differential diagnosis of ichthyosis and related ocular findings

Condition	Cutaneous Manifestations	Ocular Manifestations	Histopathology	Genetic Basis	Inheritance Pattern
X-linked Ichthyosis (XLI)	Polygonal, dark scales on extensor surfaces; sparing of palms/soles	Posterior stromal opacities, prominent corneal nerves, dry eye, rare erosions	Orthokeratosis; retained granular layer	Steroid sulfatase deficiency (STS gene, X chromosome)	X-linked recessive
Ichthyosis Vulgaris	Fine, whitish scales on extensor surfaces	Mild dry eye, rare corneal involvement	Orthokeratosis, absent granular layer	Filaggrin gene mutation (FLG)	Autosomal semi-dominant
Epidermolytic Ichthyosis	Blisters, erosions at birth, hyperkeratotic plaques	Recurrent painful corneal erosions	Epidermolytic hyperkeratosis, acantholysis	KRT1 or KRT10 mutations	Autosomal dominant
Lamellar Ichthyosis	Thick plate-like scales, widespread erythema	Potential for keratitis, corneal erosions	Orthokeratosis, normal or widened granular layer, acanthosis, papillomatosis	TGM1 or lipid-processing mutations	Autosomal recessive

XLI typically manifests in early infancy with dry, scaling skin that persists lifelong, often appearing within the first few weeks as polygonal, translucent scales that evolve into tightly adherent, dark brown scales by childhood [2]. Severity varies, with some patients showing mild scaling and others extensive coverage. Ocular involvement, such as stromal opacities, may emerge in childhood but often becomes symptomatic in adolescence, as in this case, with risks of chronic ocular surface disease and scarring if untreated [2]. Systemic features like cryptorchidism (20% prevalence) and increased testicular cancer risk may also develop, necessitating broader monitoring [2].

A multidisciplinary approach involving dermatologists and ophthalmologists was crucial in diagnosing and managing this patient’s condition. The involvement of the eyelids in ichthyosis can lead to a tightening of the skin and lagophthalmos, which can exacerbate ocular surface stress. Frequent lubrication and therapies to address lagophthalmos are essential to manage these complications. In ichthyosis, management of ocular surface disease typically involves aggressive lubrication with preservative-free artificial tears and ointments [11]. In cases with significant inflammation, anti-inflammatory agents such as topical cyclosporine or lifitegrast may be considered [12]. Consensus recommendations for retinoid use in ichthyosis support systemic retinoids like acitretin for severe scaling and topical retinoids like tazarotene for localized issues such as eyelid complications, tailored to pediatric and adolescent patients to minimize side effects from topical keratolytics (e.g., urea, salicylic acid) [13]. In severe cases, surgical interventions may be required to correct eyelid tightness or incomplete closure. Surgical options for lagophthalmos in ichthyosis may include skin grafts or flaps to release tight skin, or procedures to improve eyelid closure, such as lateral tarsorrhaphy [14].

While ichthyosis can present with various ocular surface manifestations, early recognition, and treatment are key to preventing long-term complications such as corneal scarring and vision loss. Addressing ichthyosis's cutaneous and ocular manifestations can improve patients' outcomes and quality of life [1,15]. Given the condition's inheritable nature, genetic counseling is an important aspect of the multidisciplinary approach, and this is important for informing families about recurrence risks and reproductive options.

## Conclusions

This case underscores the critical interplay between systemic skin disorders, such as XLI, and ocular surface health, emphasizing the importance of early recognition and a multidisciplinary approach to care. Initially misdiagnosed as eczema, the patient’s ichthyosis manifested with ocular findings including recurrent corneal erosions (likely resulting from abnormalities in the corneal basement membrane) and deep stromal opacities. These findings highlight the diagnostic challenges posed by overlapping dermatological and ocular symptoms and the potential consequences of delayed or inaccurate diagnosis.

By integrating dermatology and ophthalmology in both diagnosis and management, clinicians can address the systemic nature of conditions like XLI more effectively, preventing long-term complications such as vision impairment and corneal scarring. This case demonstrates how a collaborative approach not only improves diagnostic accuracy but also enhances patient outcomes and quality of life by mitigating morbidity associated with these underrecognized systemic disorders.
